# Atomically isolated nickel species anchored on graphitized carbon for efficient hydrogen evolution electrocatalysis

**DOI:** 10.1038/ncomms10667

**Published:** 2016-02-10

**Authors:** Lili Fan, Peng Fei Liu, Xuecheng Yan, Lin Gu, Zhen Zhong Yang, Hua Gui Yang, Shilun Qiu, Xiangdong Yao

**Affiliations:** 1 School of Natural Sciences, Queensland Micro- and Nanotechnology Centre, Griffith University, Brisbane, QLD 4111, Australia; 2 State Key Laboratory of Inorganic Synthesis and Preparative Chemistry, College of Chemistry, Jilin University, Changchun 130012, China; 3 Key Laboratory for Ultrafine Materials of Ministry of Education, School of Materials Science and Engineering, East China University of Science and Technology, Shanghai 200237, China; 4 Institute of Physics, Beijing National Laboratory for Condensed Matter Physics, Chinese Academy of Sciences, Beijing 100190, China

## Abstract

Hydrogen production through electrochemical process is at the heart of key renewable energy technologies including water splitting and hydrogen fuel cells. Despite tremendous efforts, exploring cheap, efficient and durable electrocatalysts for hydrogen evolution still remains as a great challenge. Here we synthesize a nickel–carbon-based catalyst, from carbonization of metal-organic frameworks, to replace currently best-known platinum-based materials for electrocatalytic hydrogen evolution. This nickel-carbon-based catalyst can be activated to obtain isolated nickel atoms on the graphitic carbon support when applying electrochemical potential, exhibiting highly efficient hydrogen evolution performance with high exchange current density of 1.2 mA cm^−2^ and impressive durability. This work may enable new opportunities for designing and tuning properties of electrocatalysts at atomic scale for large-scale water electrolysis.

## Abstract (web summary)

There is tremendous ongoing effort in the development of electrocatalysts for hydrogen evolution. Here, the authors report that single nickel atoms dispersed on graphitic supports are formed by carbonization of metal-organic frameworks and that they are highly active hydrogen evolution catalysts.

## Graphical abstract

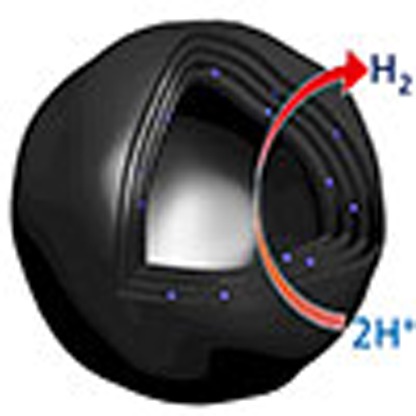

Molecular hydrogen (H_2_), when generated from carbon-neutral processes, plays a vital role in the sustainable energy systems and chemical industry^1^,^2^,^3^. Electrolysis has stood out among various hydrogen production technologies because of high energy-conversion efficiency and environmentally benign process, especially coupled with energy sources such as solar and wind^4^,^5^,^6^. In this rapidly developed field, finding active catalysts for hydrogen evolution reaction (HER) to lower large potentials is of paramount importance to promote water electrolysis application. It has been confirmed that platinum (Pt) is the most active and stable electrocatalyst for HER, with only small overpotentials for high reaction rate. However, its low abundance and consequently high cost limit its large-scale commercial applications. Hence, exploring cheap, efficient and durable alternatives of Pt is crucial to facilitate the global scalability of such potential clean-energy technology^7^. Therefore, a series of Pt-free electrocatalysts have been identified for HER in strong acids, such as metal compounds^8^,^9^,^10^,^11^,^12^,^13^,^14^,^15^,^16^,^17^ (sulfides, nitrides, carbides, borides and phosphides) and carbon-based materials^18^,^19^,^20^. Moreover, remarkable advances in nanostructuring or stabilizing these electrocatalysts on carbon substrates have further exposed more active sites simultaneously with enhanced electrical conductivity^21^,^22^,^23^,^24^,^25^,^26^,^27^. Recently, design strategies like encapsulating 3d transition metals into carbon nanotubes and graphene nanoshells have been reported to improve the long-term activity^28^,^29^,^30^. Even so, compared with Pt, these reported materials show dwarfed in activity and durability.

Isolated metal atoms, owing to their low-coordination and unsaturated atoms functioned as active sites, have been demonstrated more catalytic active than nanometer-sized metal particles^31^,^32^,^33^,^34^,^35^. Supported isolated noble metals, such as palladium, Pt and gold, have been reported as highly active catalysts for hydrogenation reactions, CO oxidation reactions and water–gas shift reactions^33^,^34^,^35^. However, earth-abundant 3d transition metals, which have been investigated as promising alternatives in alkaline electrolytes^36^,^37^, have not been downsized to single atoms to improve their activity for HER so far.

Herein, we show a nickel–carbon (Ni–C)-based catalyst, which can be tuned by electrochemical methods to obtain atomically isolated Ni species anchored on graphitized carbon, consequently displaying high activity and durability for HER. The activated-Ni–Carbon (A-Ni–C) catalyst exhibts overpotential for the current density of 10 mA cm^−2^, 20 mA cm^−2^ and 100 mA cm^−2^ at −34 mV, −48 mV and −112 mV, respectively, a low Tafel slope of 41 mV per decade and a large exchange current density of 1.2 mA cm^−2^. More importantly, A-Ni–C could maintain high activity for >25 h in the chronoamperometric test. A variety of analytical techniques illustrate that Ni single atoms formed during the activation process, which could contribute greatly to HER performance. Compared with Pt and other Pt alternatives, A-Ni–C is composed of more abundant elements in the earth's crust. These findings in our work may help accelerate the large-scale application of proton exchange membrane (PEM) electrolysers and solar photoelectrochemical (PEC) water electrolysers.

## Results

### Sythesis and characteration of A-Ni–C catalyst

**Figure 1:**
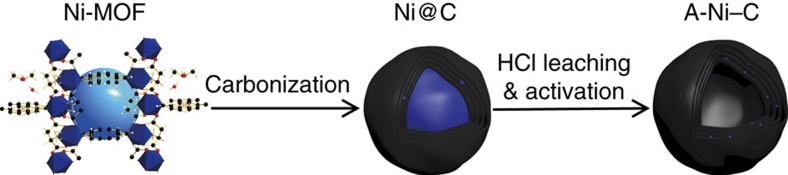
Schematic diagram of synthesis and activation process of the Ni–C catalysts. The Ni-MOF used as a precursor consists of an orthorhombic crystal. Atoms are shown as follows: C, black; H, white; O, red; N, blue; Ni, royal blue. Carbonization of the synthesized Ni-MOF was at 700 °C in N_2_ atmosphere to obtain Ni@C. HCl leaching treatment was repeated three times to sufficiently dissolve exposed Ni metal. Constant potential and CV treatments were performed to activate the catalysts until they reached the optimal performance and remained stable. During the activation process, Ni single atoms formed *in situ* anchored on the graphitized carbon.

A-Ni–C catalyst was obtained from well-dispersed Ni metal in the graphitized carbon matrix. The synthesized Ni-based metal-organic framework (Ni-MOF) was used as the precursor (Fig. 1). Followed by carbonization at 700 °C in nitrogen (N_2_) atmosphere, Ni nanoparticles encapsulated in graphene layers (Ni@C) were prepared. Hydrochloric acid (HCl) leaching treatment was used to remove the redundant Ni metal (HCl-Ni@C). After HCl treatment, hollow onion-like carbon nanoshells along with little Ni nanoparticles protected by graphene layers remained. We then applied electrochemical cyclic-potential on the HCl-Ni@C catalysts-decorated electrode, unexpected activation process was observed. Similar activation processes were also reported in the literature^13^,^20^. To our surprise, direct constant potential on Ni@C can also activate the electrocatalyst, which was conducted until the electrocatalyst reached the optimized and stable activity. We found Ni single atoms formed during all these activation process, which is verified as discussed later.

**Figure 2:**
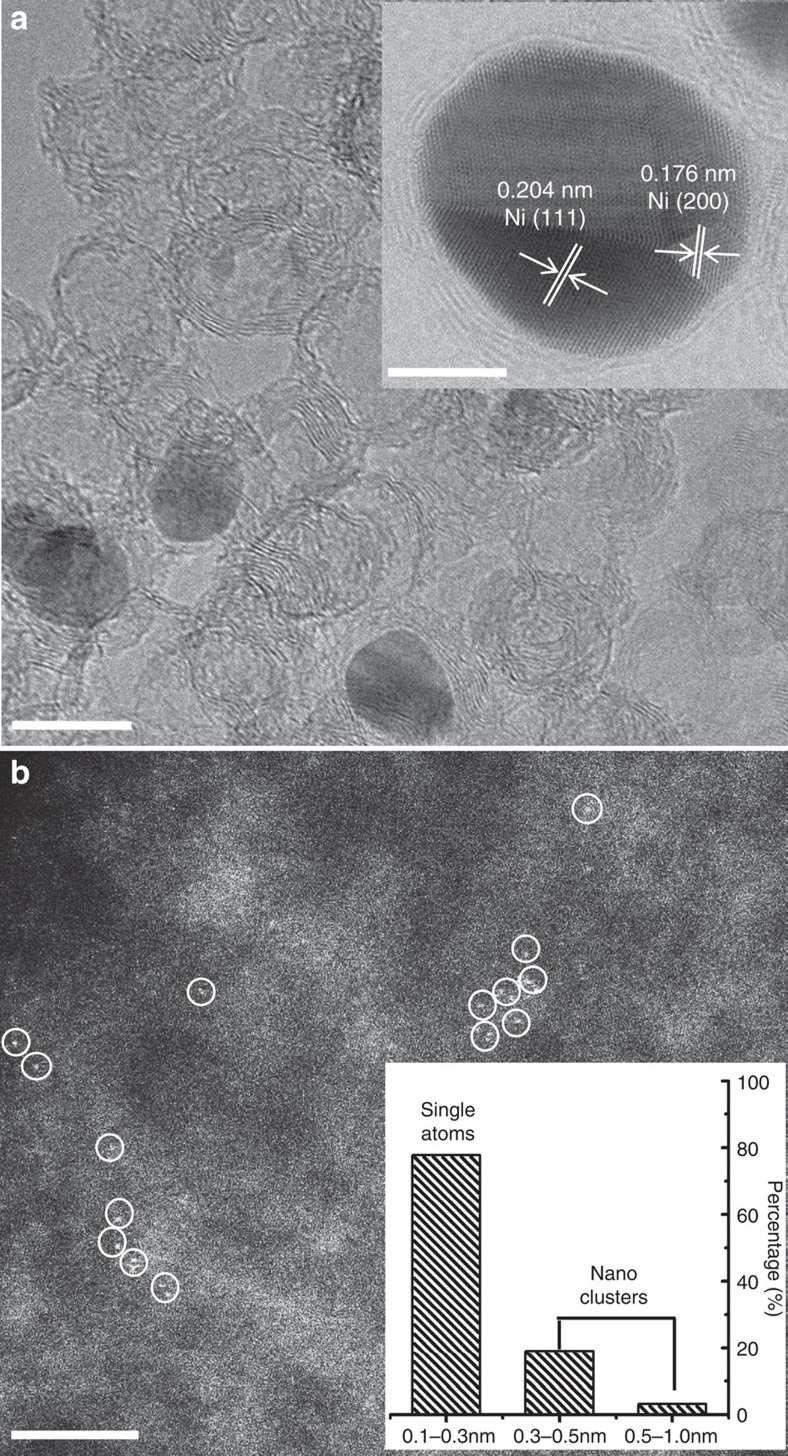
Micrographs of samples HCl-Ni@C and A-Ni–C. (**a**) HRTEM image of HCl-Ni@C. Scale bar, 10 nm. Inset of **a**, BF STEM image of Ni metal encapsulated in graphene layers. Scale bar, 5 nm. The area of graphene with exposure of Ni nanoparticle provides the opportunity that Ni metal would slowly resolve during the activation process. (**b**) Subångström resolution HAADF STEM image of A-Ni–C. The circles are drawn around isolated Ni atoms. Scale bar, 3 nm. Inset of **b**, the size distribution figure, which is based on >120 observed Ni species counted from high-magnification images (recorded at 4 × original magnification).

The nanostructure of Ni@C was characterized by a transmission electron microscope (TEM). Low-magnification TEM image of Ni@C displays Ni metal inside carbon nanospheres (Supplementary Fig. 1a). The size distribution image (Supplementary Fig. 1b) suggests that the nanospheres have an average size around 10 nm. After HCl leaching, the high-resolution TEM image of HCl-Ni@C shows hollow onion-like nanoshells along with little Ni metal aggregates encapsulated in the graphene layers (Fig. 2a). To further study the structure changes of the catalyst before and after activation, a JEM-ARM200F scanning TEM (STEM) fitted with a double aberration-corrector for both probe-forming and the imaging lenses was used. In the STEM image of HCl-Ni@C, the measured Ni (111) d-spacing (0.204 nm) and Ni (200) d-spacing (0.176 nm) again confirm the existence of well crystalline Ni metal in the graphitic carbon nanoshells (inset of Fig. 2a). The thickness of graphene layer is uneven with exposure of Ni nanoparticles, which would provide possibilities for slow dissolution of Ni metal during the activation process in the acidic solution. The bright-field STEM image of A-Ni–C (Supplementary Fig. 2) illustrates that the measured (002) d-spacing of the graphitic carbon ranges from about 0.33 to 0.36 nm, indicating the carbon structure is somewhat disordered, rather than ideally graphitic, which was also evidenced by the X-ray diffraction (XRD) and Raman analysis. Individual metal atoms in practical catalysts can be discerned in the atomic resolution high-angle annular dark-field (HAADF) images^33^,^34^,^35^. For the sample A-Ni–C, the atomically isolated Ni species (marked by white circles) are dispersed on the partially graphitized carbon matrix (Fig. 2b). Examination of different regions (Supplementary Fig. 2) confirms abundance of Ni single atoms present in A-Ni–C. Considering that the elements of N, F and S have a smaller atomic number than Ni, these impuries can hardly exsit as bright tiny spots in the metal-carbon system. HAADF images of controled samples have also ruled out this possiblity (Supplementary Fig. 3). About 78% of the Ni species counted in the images were present as single atoms away from each other (inset of Fig. 2b). A minority of subnanometer Ni clusters present in the same sample did not have the packed Ni atom structure of Ni nanoparticles.

**Figure 3:**
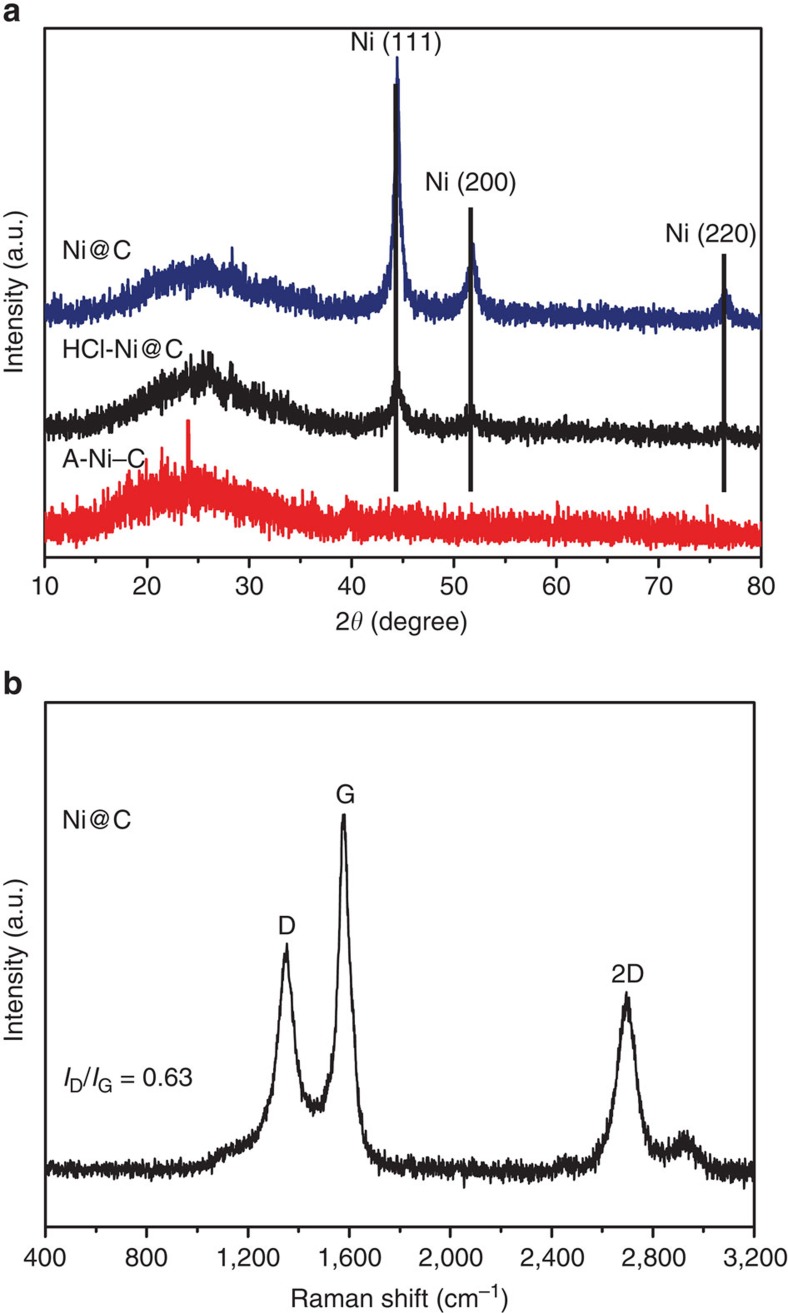
Structure characterization of the Ni–C catalysts. (**a**) XRD patterns of Ni@C, HCl-Ni@C and A-Ni–C, respectively. The diffraction peaks of Ni in Ni@C and HCl-Ni@C confirm the existence of well crystalline Ni metal. The red line corresponding to A-Ni–C illustrates that no Ni–Containing crystal phase was detected after activation process. (**b**) Raman spectrum of Ni@C with *I*_D_/*I*_G_=0.63, indicating the partial graphitization at 700 °C.

The multiple peaks on the XRD pattern of the synthesized Ni-MOF (Supplementary Fig. 4a) well match those of the typical crystalline structure of [Ni_2_(L-asp)_2_(bpy)]·CH_3_OH·H_2_O. Ni@C exhibits the cubic phase of Ni metal (JSPDS NO. 04-0850) with a broad shoulder peak in the range 20–30° (2*θ*), which originates from the partially graphitized carbon specie (Fig. 3a). The diffraction peaks of graphitic carbon become higher and sharper with the increased temperature (Supplementary Fig. 4b). Raman spectrum of Ni@C (Fig. 3b) further confirms that the Ni-MOF is partially graphitized at 700 °C. Among carbon-based catalysts, these graphitic carbon structures formed during the carbonization treatment may significantly enhance the electronic conductivity and corrosion resistance, which would improve the electrocatalytic efficiency^29^. After sufficiently washed by HCl, little Ni nanoparticles encapsulated in the multi graphene layers remained. The XRD pattern of HCl-Ni@C proves the existence of the Ni metal. However, no Ni–Containing signal was detected in the sample A-Ni–C, primarily attributed to insensitivity of XRD to single atoms or small clusters.

**Figure 4:**
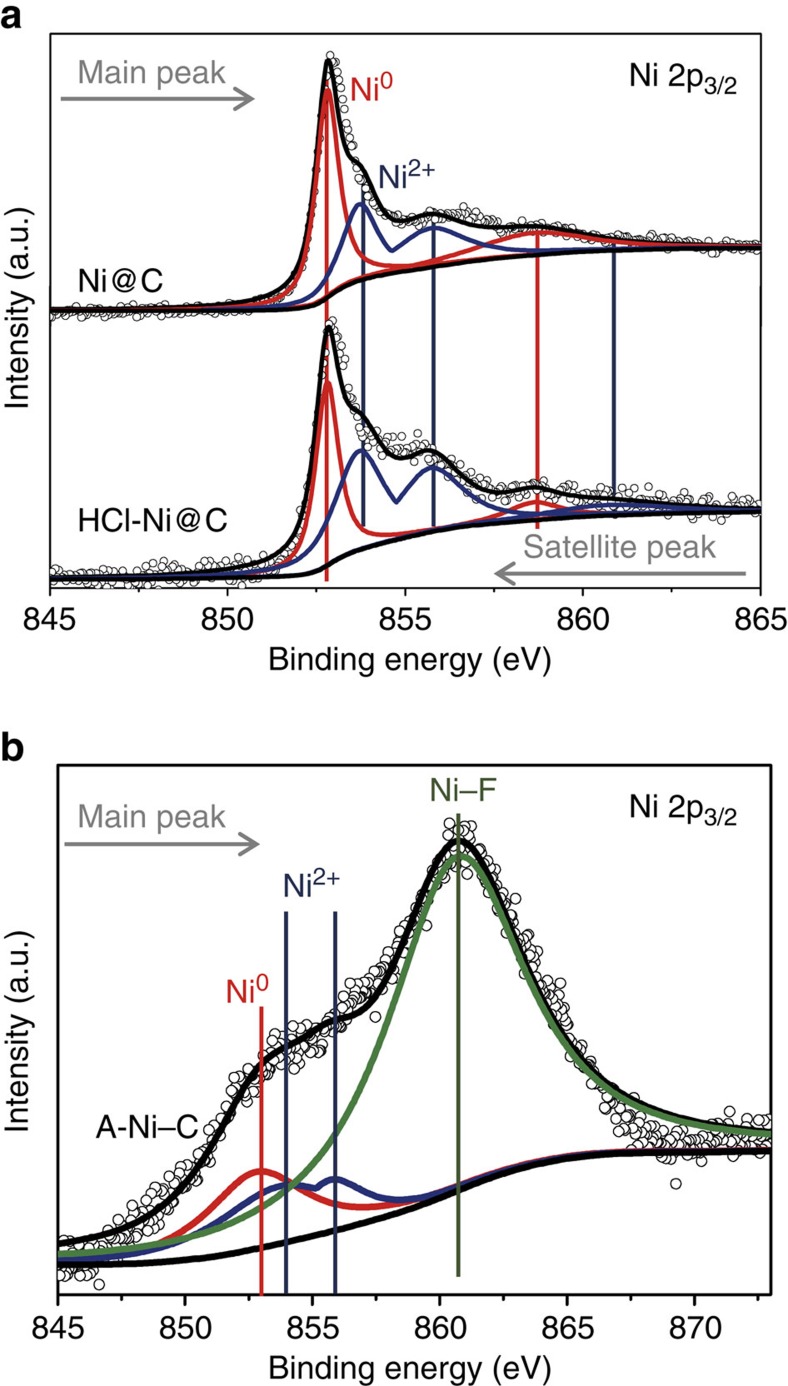
XPS spectra of the Ni–C catalysts. (**a**) XPS spectra of Ni 2p peaks of Ni@C and HCl-Ni@C. (**b**) XPS spectrum of Ni 2p peaks of A-Ni–C. The peaks at 852.8 eV and 853.7 eV are assigned to Ni^0^ and Ni^2+^, respectively. The sharp peak at 860.7 eV can be correlated with Ni–F binding, and the element F comes from Nafion solution.

The binding state was investigated by X-ray photoelectron spectroscopy (XPS). As shown in Supplementary Fig. 5, N–C bonds in the samples of Ni@C, HCl-Ni@C and A-Ni–C are in the form of pyridine (398.9 eV), pyrrole (400.4 eV) and graphite (401.3 eV) (refs 28,38). The quantitative analysis of the XPS results reveals that the atomic ratios of N/C in Ni@C, HCl-Ni@C and A-Ni–C are 4.62:100, 3.80:100 and 2.21:100, respectively. The existence of N dopants in the A-Ni–C would inherently increase the ability of interacting with reactants and render high positive charge density on their adjacent carbon atoms, which could also contribute to the high activity of A-Ni–C samples^28^,^29^. The Ni 2p XPS spectrum of Ni@C reveals the coexistence of Ni^0^ (852.8 eV) and Ni^2+^ (853.7 eV), which can be ascribed to Ni-Ni and Ni-O bonding^39^,^40^ (Fig. 4a). After HCl leaching, the Ni 2p XPS spectrum of HCl-Ni@C nearly remains the same as that of Ni@C, suggesting the residue of Ni metal. The existence of Ni-O bonding can be attributed to the slow oxidation of the exposed Ni metal^41^,^42^,^43^. We further collected samples of A-Ni–C for analysis. The XPS analysis of A-Ni–C (Supplementary Fig. 6, Supplementary Table 1) illustrates it contains Ni, C, N, F, S and O elements without any Pt impurity, eliminating the influence of Pt for HER performance. We should point out that F, S and O elements mainly resulted from the Nafion solution. To confirm that trace amount of Ni in the A-Ni–C sample contributes to the HER activity, we determined the Ni contents by indicatively coupled plasma atomic emission spectroscopy. As shown in Supplementary Table 2, the weight ratios of Ni in the Ni@C, HCl-Ni@C and A-Ni–C were 85.0%, 5.3% and 1.5%, respectively. We found that the Ni content decreased during the CV activation process. What is more, no detectable Pt impurities were found in all these samples. In addition, the Ni 2p XPS spectrum of A-Ni–C (Fig. 4b) has a sharp peak ∼860.7 eV, which can be associated with the strong Ni–F binding. Specifically, the peak at 852.8 eV is assigned to Ni^0^, reflecting Ni–Ni binding of the Ni clusters.

### Electrocatalytic HER activity evaluation

**Figure 5:**
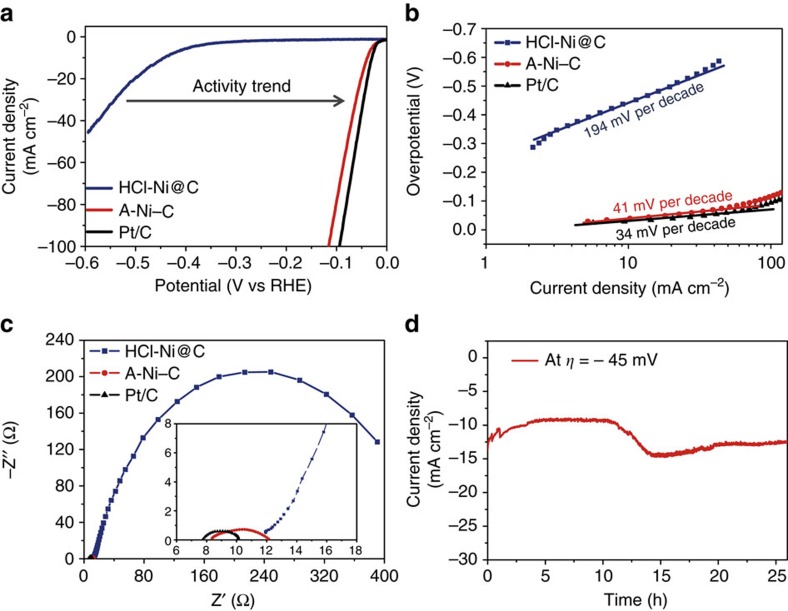
HER electrocatalytic properties of the Ni–C catalysts. (**a**) LSV curves of HCl-Ni@C, A-Ni–C and Pt/C. (**b**) Tafel plots obtained from LSV curves in **a**, indicating significant improvement in activity for HER after activation. (**c**) EIS spectra of HCl-Ni@C, A-Ni–C and Pt/C at the overpotential of −100 mV. Inset of **c** represents a close-up view of the spectra at high frequencies. A-Ni–C-modified GC electrode displays much lower impedance than HCl-Ni@C-modified GC electrode, similar to that of Pt/C-modified GC electrode. (**d**) Chronoamperometric curve of A-Ni–C at *η*=−45 mV, revealing nearly no apparent deactivation after 25 h. Note: all the electrochemical data of A-Ni–C catalysts were obtained after electrochemical cyclic-potential activation.

The HER electrocatalytic activity was evaluated in 0.5 M H_2_SO_4_ using a typical three-electrode set-up. All tests were performed without *iR* compensation. The lineal sweep voltammetry (LSV) curves showing the normalized current density (*j*) versus voltage (*j* versus *V*) for A-Ni–C and HCl-Ni@C along with commercial Pt/C (5 wt% Pt on Vulcan carbon black), for comparison, are show in Fig. 5a. As expected, Pt/C catalyst exhibits excellent HER performance. HCl-Ni@C requires overpotential (*η*) of about −440 mV to reach *j* of 10 mA cm^−2^. In sharp contrast, A-Ni–C produces *j* of 10 mA cm^−2^, 20 mA cm^−2^ and 100 mA cm^−2^ at *η* of −34 mV, −48 mV and −112 mV, respectively. These overpotentials are the lowest among the reported acid-stable and earth-abundant HER electrocatalysts, including recently reported Ni_2_P^14^, MoP | S^16^, FeP^17^, MoS_2_ (refs 8,22,24), WS_2_ (refs 21,23,25), Mo_2_C (refs 12,13) and chemically doped grapheme-based materials^18^,^19^,^20^ (more details in Supplementary Table 3).

To understand the detailed underlying mechanism of HER activity, Tafel plots based on LSV curves were acquired (Fig. 5b). The linear regions of Tafel plots were fit to the Tafel equation (*η*=*b* log *j*+*a*, where *b* is the Tafel slope), yielding Tafel slopes of ∼34, 41, 194 mV per decade for Pt/C, A-Ni–C and HCl-Ni@C, respectively. The value for A-Ni–C does not correspond to one of the standard HER Tafel slopes (29, 38, 116 mV per decade), indicating the HER occurs through a Volmer–Heyrovsky mechanism and electrochemical recombination with an additional proton is the rate-limiting step^28^. Moreover, the exchange current density (*j*_0_) was obtained by extrapolation of Tafel plots (Supplementary Fig. 7). The *j*_0_ of A-Ni–C is about 1.2 mA cm^−2^ (the same magnitude as the value of 2.5 mA cm^−2^ for Pt/C), also highlighting the exceptional H_2_ evolving efficiency of A-Ni–C catalyst.

The optimal activity of A-Ni–C catalyst would also be attributed to strong chemical and electronic coupling between graphitized carbon and Ni single atoms, permitting highly efficient electrical communication between the catalytic active sites and the underlying electrode substrate. Electrochemical impedance spectroscopy measurements were performed to confirm the hypothesis (Fig. 5c). The charge transfer resistance of A-Ni–C is similar to that of Pt/C, which is much lower than that of HCl-Ni@C. Thus, much faster electron transfer between the catalytic active sites of A-Ni–C and the electrode substrate is one of the key factors contributing to the superior HER kinetics.

### Durability of A-Ni–C catalyst

The durability is a key concern of all catalysts. In this study, accelerated degradation measurements were adopted to evaluate the durability. Cyclic voltammetric (CV) sweeps between+905 mV and −95 mV versus the reversible hydrogen electrode potential (versus RHE) at 100 mV s^−1^ were applied to HCl-Ni@C-decorated glassy carbon (GC) electrode. Post-potential-cycling LSV curves were recorded (Supplementary Fig. 8). HER performance after 8,000 cycles retained almost the same to the test after 4,000 cycles. To further probe the durability of A-Ni–C in the acidic environment, continuous HER at a static overpotential was conducted (Fig. 5d). When operating a constant overpotential of −45 mV, a continuous HER process occurred to generate molecular H_2_ (different overpotentials were applied to generate H_2_ shown in Supplementary Movie 1). During the first 11 h, the current density of ∼10 mA cm^−2^ was observed, and then gradually increased to ∼14 mA cm^−2^, which might be caused by adequate activation of the catalyst. In addition, the current density levelled out at ∼12 mA cm^−2^. It should be mentioned that the high activity was maintained for >25 h in the chronoamperometric electrolysis (after CV activation, ⩾4,000 cycles). Furthermore, we did the galvanostatic electrolysis test to collect the evolved hydrogen gas to evaluate the Faradaic efficiency of the A-Ni–C catalyst. In Supplementary Fig. 9, the result demonstrates that A-Ni–C gives about 100% Faradaic yield during the electrolysis process. Overall, the high catalytic activity and outstanding durability illustrate the potential of A-Ni–C for cost-effective electrocatalytic hydrogen evolution in water electrolysis system and solar-driven hydrogen system.

### Activation processes

**Figure 6:**
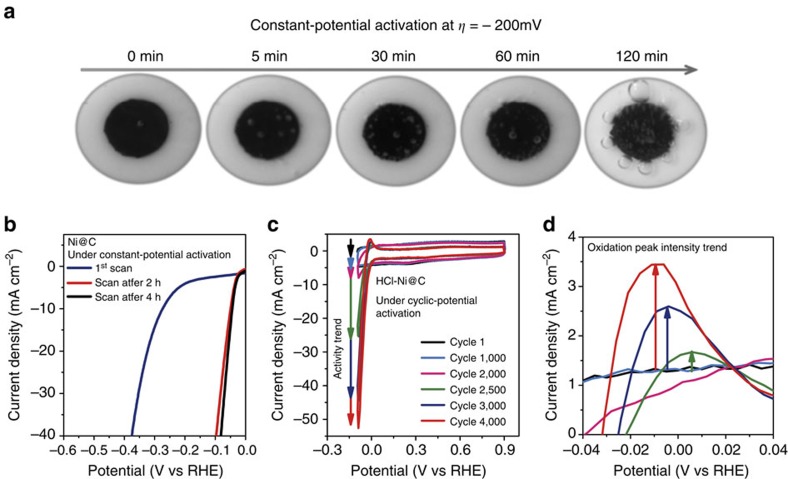
Activation processes of the Ni–C catalysts. (**a**) Hydrogen bubbles driven at *η*=−100 mV at GC electrodes with different degrees of activation (constant-potential activation at *η*=−200 mV for different time). All the digital photos were taken when the cell turned on for 20 s. The diameter of glassy carbon electrode is 5 mm. (**b**) Initial and post-constant-potential activation LSV curves of Ni@C. The applied constant potential would activate Ni@C catalyst. (**c**) Cyclic voltammograms of HCl-Ni@C (cycle 1, cycle 1,000, cycle 2,000, cycle 2,500, cycle 3,000 and cycle 4,000 were selected). Catalytic activity trend corresponds to the improving current density. (**d**) Oxidation peaks of the catalyst in different cycles. The oxidation peak intensity increased and the peak potentials shifted in the negative direction in the anodic-going scan.

Activation processes (constant-potential activation and cyclic-potential activation) were also recorded to investigate changes after activation. Hydrogen bubbles generated at *η*=−100 mV on Ni@C-decorated GC electrodes with different degrees of activation (Fig. 6a). Constant potential at *η* of −200 mV (Supplementary Fig. 10) was used to stabilize the catalyst to reach the best performance, simultaneously ruling out the possibility that the high *j* would result from the reaction between the unsecure Ni metal and the strongly acidic solution. The *j* gradually improved in the first 2 h and levelled out in the next 2 h. These activation treatments also ensured stable and reproducible results. LSV curves of Ni@C after different periods of constant-potential activation prove the activation process of Ni@C (Fig. 6b). During CV activation process, the catalytic *j* of HCl-Ni@C gradually increased with the consecutive CV scans and reached the maximum efficiency after about 4,000 cycles (Fig. 6c). Notably, the anodic peaks around 0 V (versus RHE) shifted to lower potentials during the process, indicating a more easily occurring oxidation reaction. Interestingly, the oxidation peak intensities were also enhanced during the CV treatment (Fig. 6d). These signs during CV activation reveal that A-Ni–C could lose electrons more easily in the anodic-going scan. The oxidation peak intensity gradually levelled out when the cathodic catalytic *j* nearly remained stable (Supplementary Fig. 11). These changes of the oxidation peak corresponding to valance changes of Ni coincide with the trend of the catalytic activity. In addition, we also collected the activated samples after different CV cycles for STEM analysis (Supplementary Figs 12 and 13). The downsizing of the Ni nanoparticles and the formation of single Ni atoms can be evident to be observed during the activation process. Throughout the detailed CV activation process, we summarize that active Ni single atoms would form during the activation process, leading to these changes of the oxidation peak and simultaneously contributing greatly to the improved activity. Furthermore, the HCl-Ni@C samples can also be activated on the fluorine-doped SnO_2_ (FTO) glass substrate (Supplementary Figs 14 and 15).

## Discussion

In summary, we have used carbonization of MOFs to synthesize Ni–C-based catalyst and applied electrochemical potential to activate Ni–C catalyst, which can reach outstanding performance during HER processes. The discovery of this A-Ni–C catalyst highlights a new area of tuning structure and functionality of metal-carbon-based catalyst at atomic scale by electrochemical methods, which would hold the promise for large-scale real-world water splitting electrolysers.

## Methods

### Chemicals

Nickel (II) carbonate basic hydrate (48–50% Ni, Fluka), L-aspartic acid (98%, Sigma-Aldrich), 4,4′-bipyridyl (98%, Alfa Aesar), Nafion-117 solution (5 wt. % in a mixture of lower aliphatic alcohols and water) (Sigma-Aldrich), sulphuric acid (98%, Merck) and HCl (32%, RCI labscan) were used as received without any further purification.

### Synthesis of Ni(L-asp)(H_2_O)_2_·H_2_O

Measured amount of NiCO_3_·2Ni(OH)_2_· × H_2_O (2.450 g) and L-aspartic acid (2.630 g) were dissolved in water (200 ml) under stirring and heating for 3 h. After filtering out all the remaining solid particles, the clear turquoise solution was then evaporated at 80 °C oven overnight to obtain the pale blue Ni(Asp) powder.

### Synthesis of [Ni_2_(L-asp)_2_(bpy)]·CH_3_OH·H_2_O (Ni-MOF)

Ni(L-asp)(H_2_O)_2_·H_2_O (0.219 g) and 4,4′-bipyridyl (0.312 g) were dispersed in a mixture containing water (6 mL) and methanol (4.742 g) under ultrasonic vibration. The final solution was sealed into an autoclave and heated at 150 °C for 48 h. The product, [Ni_2_(L-asp)_2_(bpy)]·CH_3_OH·H_2_O, was filtered, washed with copious amounts of water and methanol and dried at 60 °C for 24 h.

### Carbonization of Ni-MOF

Carbonization processes were carried out in N_2_ atmosphere. The dried Ni-MOF powder was pretreated at 150 °C for 2 h and then carbonized at different temperatures for 5 h with a heating rate of 5 °C min^−1^.

### HCl leaching treatment

Excess concentrated HCl was slowly added into Ni@C hybrids and then the suspension was put under ultrasonic condition to dissolve Ni metal. The suspension was separated when being left to stand for 6 h. The entire process was repeated two additional times. Then the product was washed with copious amount of water until the pH of the solution was ∼7 and then dried at 60 °C for 24 h.

### Electrochemical measurements

All the electrochemical tests were performed in a conventional three-electrode system at an electrochemical station (CHI 660E), using Ag/AgCl (3.5 M KCl solution) electrode as the reference electrode, Pt mesh as the counter electrode and GC electrode as the working electrode. About 4 mg of sample and 80 μl of 5 wt% Nafion solution were dispersed in 1 ml of 4:1 v/v water/ethanol by at least 30-min sonication to form a homogeneous solution. Then 5 μl or 15 μl of the solution was loaded onto the GC electrode of 3 mm or 5 mm in diameter, respectively. The final loading for all catalysts and commercial Pt/C electrocatalysts on the GC electrodes is about 0.283 mg cm^−2^. Similar preparation of titanium foil electrode was conducted with the same loading of the HCl-Ni@C samples and corresponding chronoamperometric curves of A-Ni–C was shown in Supplementary Fig. 16. LSV with scan rate of 5 mV s^−1^ was conducted in 0.5 M H_2_SO_4_ deaerated with argon at room temperature. The Ag/AgCl/3.5 M KCl reference electrode was calibrated with respect to RHE. The calibration was performed in the high purity hydrogen saturated electrolyte with Pt mesh as the working electrode and counter electrode. CVs were run at a scan rate of 1 mV s^−1^, and the average of the two potentials at which the current crossed zero was taken to be the thermodynamic potential for the hydrogen electrode reactions (Supplementary Fig. 17). In 0.5 M H_2_SO_4_, *E*_RHE_=*E*_Ag/AgCl_+0.209 V. AC impedance measurements were carried out in the same configuration when the working electrode was biased at the overpotential of 100 mV from 10^5^ Hz to 10^−1^ Hz with an AC voltage of 5 mV. The CV measurements were performed by use of the scan rate at 100 mV s^−1^ between +905 mV and −95 mV (versus RHE) without accounting for uncompensated resistance. Chronoamperometric measurement (*η*=−45 mV) was performed to evaluate the long-term stability. The faradaic yield was calculated from the total amount of electrons passed through the cell during the galvanostatic electrolysis and the amount of the evolved H_2_ gas collected by the water drainage method. The theoretically expected amount of H_2_ was calculated by applying the Faraday law, which states that the passage of 96485.4 C causes 1 equiv. of reaction. The HER performance mentioned above was well reproducible in our laboratory. More than 10 A-Ni-C-decorated GC electrodes (after CV activation) were evaluated, giving similar activation phenomenon and negligible difference of HER performance.

### Characterization

XRD patterns were acquired at room temperature using D/MAX 2550 VB/PC. Raman spectrum was recorded on a Rennishaw InVia spectrometer with a model 100 Ramascope optical fibre instrument. XPS data were collected from ESCALAB 250Xi, and the binding energy of the C 1 s peak at 284.8 eV was taken as an internal reference. TEM images were collected from TECNAI F-30 with an acceleration voltage of 300 kV. Subångström resolution STEM images were obtained on a JEM-ARM200F STEM fitted with a double aberration-corrector for both probe-forming and the imaging lenses.

## Additional information

**How to cite this article:** Fan, L. *et al*. Atomically isolated nickel species anchored on graphitized carbon for efficient hydrogen evolution electrocatalysis. *Nat. Commun.* 7:10667 doi: 10.1038/ncomms10667 (2016).

## Supplementary Material

Supplementary Supplementary Figures 1-17, Supplementary Tables 1-3 and Supplementary References

Supplementary Movie 1 Electrocatalytic hydrogen evolution at different overpotentials. Ti foil decorated with A-Ni-C electrocatalyst was used as the working electrode driven at subsequently overpotentials of - 50 mV, - 100 mV, - 200 mV and - 300 mV. HER bubbles are clearly seen at different overpotentials.
