# Supplementary material for: Atomically isolated nickel species anchored on graphitized carbon for efficient hydrogen evolution electrocatalysis
Source: Nat Commun. 2016 Feb 10;7:10667. doi: 10.1038/ncomms10667 (PMC4749971; doi:10.1038/ncomms10667)
Supplement: Supplementary — Figures 1-17, Supplementary Tables 1-3 and Supplementary References [file ncomms10667-s1.pdf]

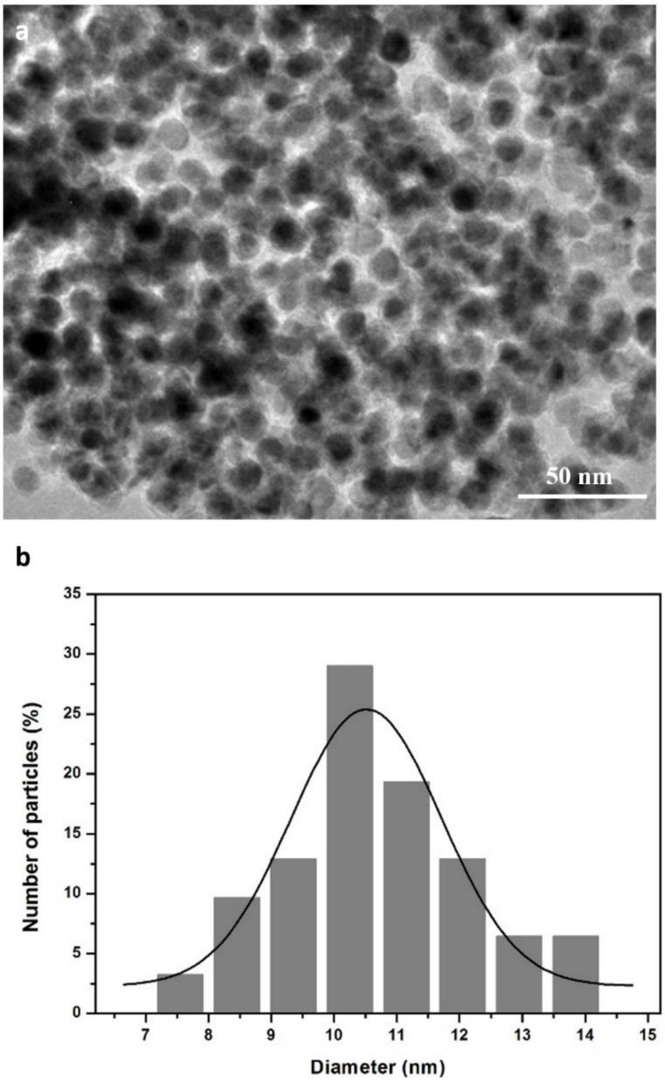

Supplementary Figure 1. (**a**) TEM image of Ni@C, displaying well-dispersed Ni in the graphitized carbon nanospheres after carbonization. (**b**) Size distribution image of Ni@C nanospheres. The size of Ni@C is around 10 nm.

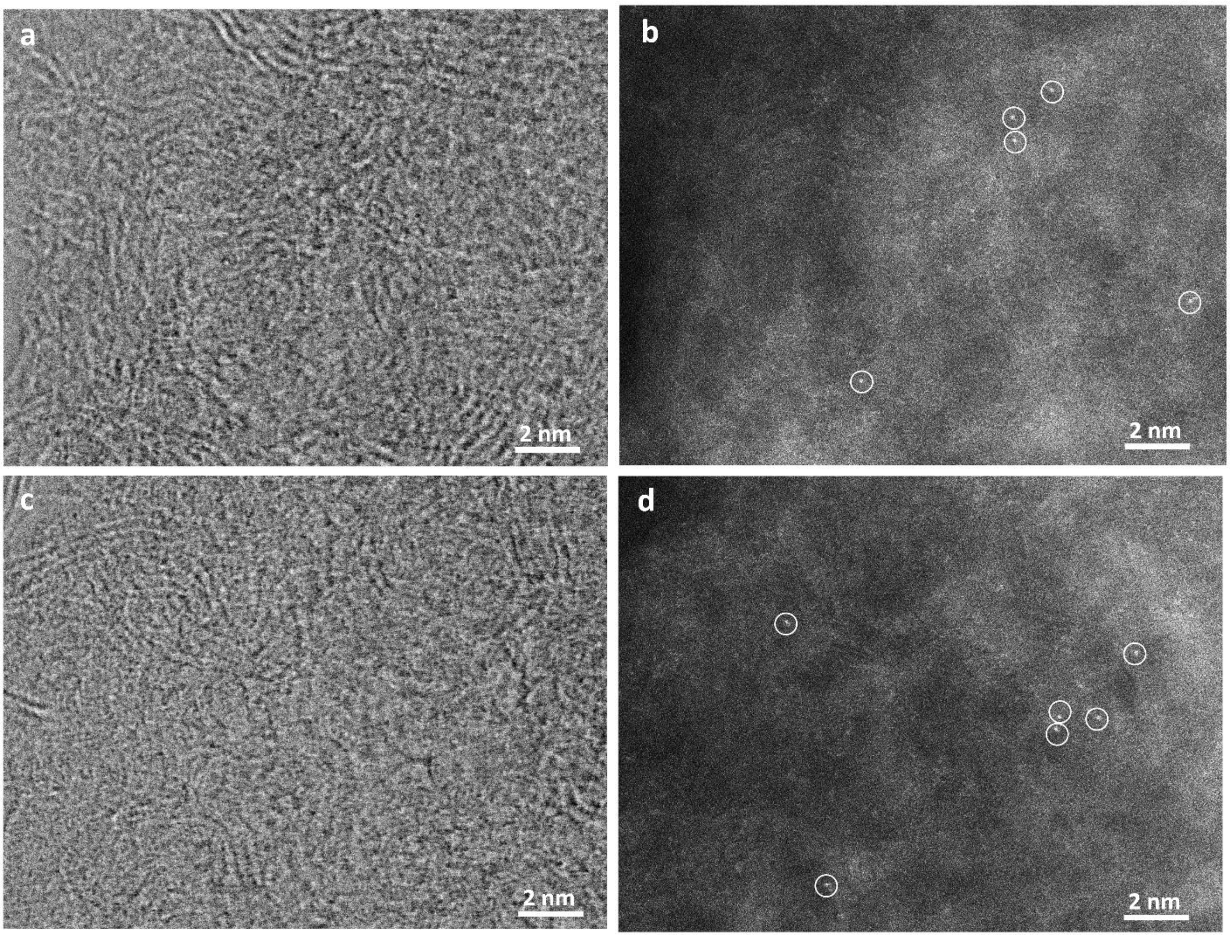

Supplementary Figure 2. A typical series of "zoom-in" STEM images of different regions of A-Ni-C. (**a**, **c**) BF STEM images and (**b**, **d**) corresponding HAADF STEM images of the same area prove abundance of atomically dispersed Ni species.

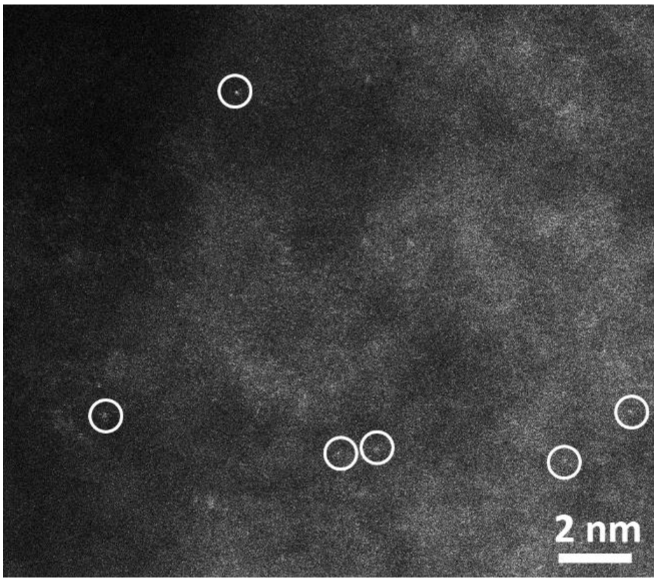

Supplementary Figure 3. HAADF STEM images of controlled samples of A-Ni-C (without Nafion as binder), showing the existence of atomically dispersed species.

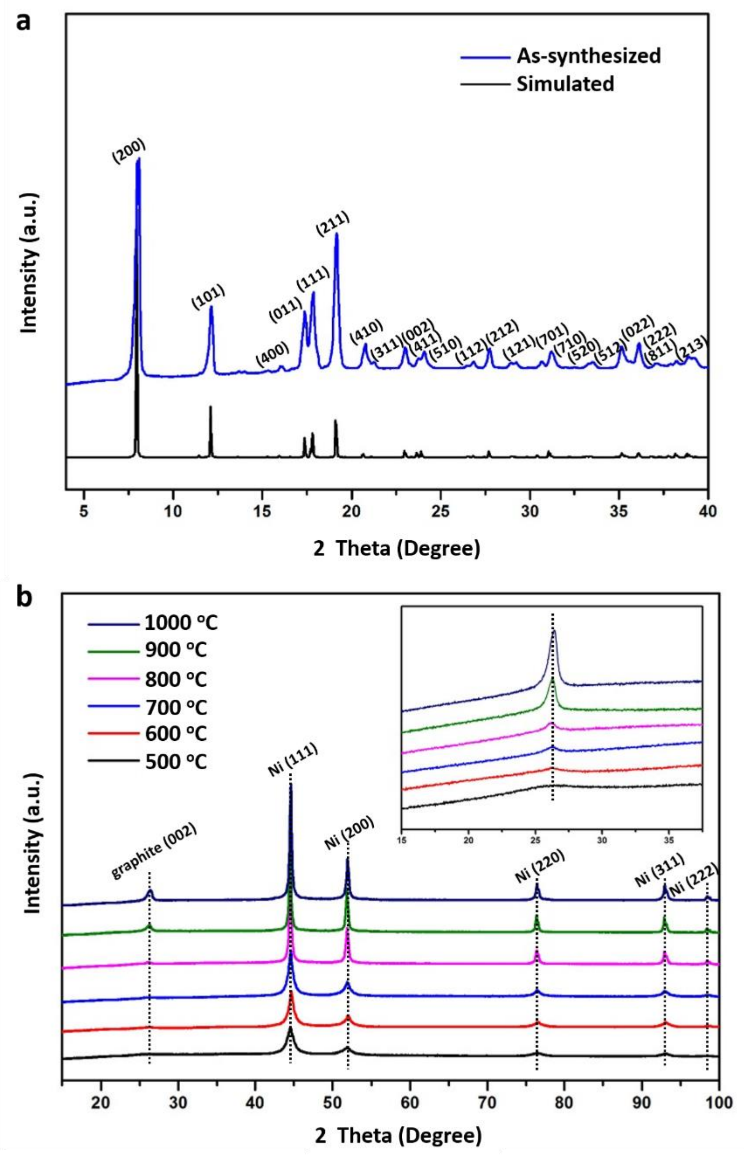

Supplementary Figure 4. (**a**, **b**) XRD patterns for (**a**) Ni-MOF and (**b**) Ni-C catalysts carbonized from Ni-MOF at different temperatures. Inset of (**b**) donates the magnified patterns in the range of 15-35°. The diffraction peaks around 26-27° attributed to graphite (002) become sharper and higher with the increased heat treatment temperature, further illustrating the increase in graphitization.

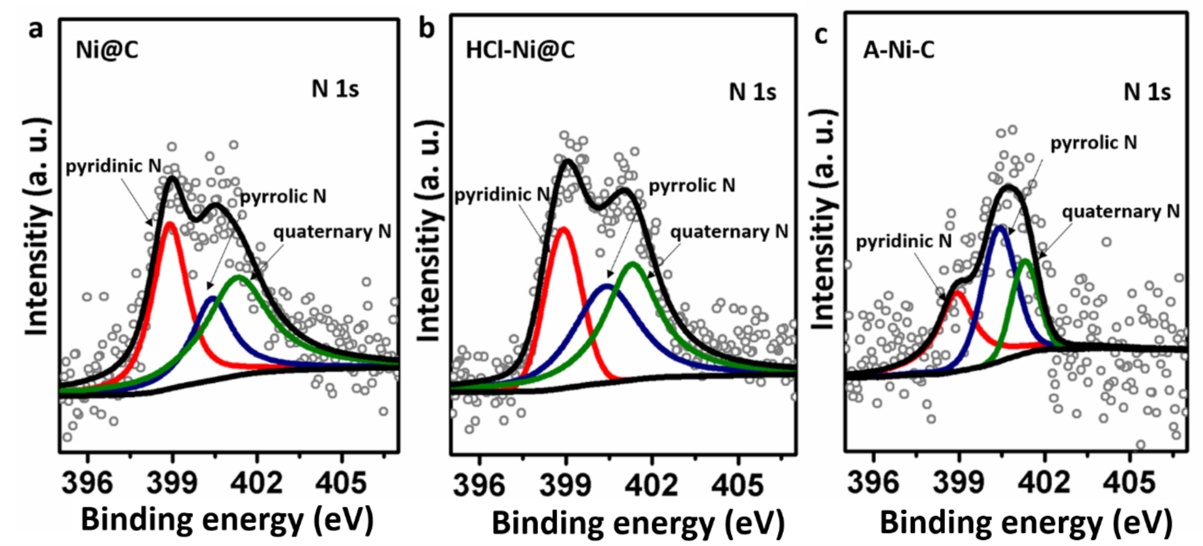

Supplementary Figure 5. N 1s core level spectra of (**a**) Ni@C, (**b**) HCl-Ni@C and (**c**) A-Ni-C, respectively, showing the presence of three types of N species in all these samples: pyridinic, pyrrolic and quaternary nitrogen atoms.

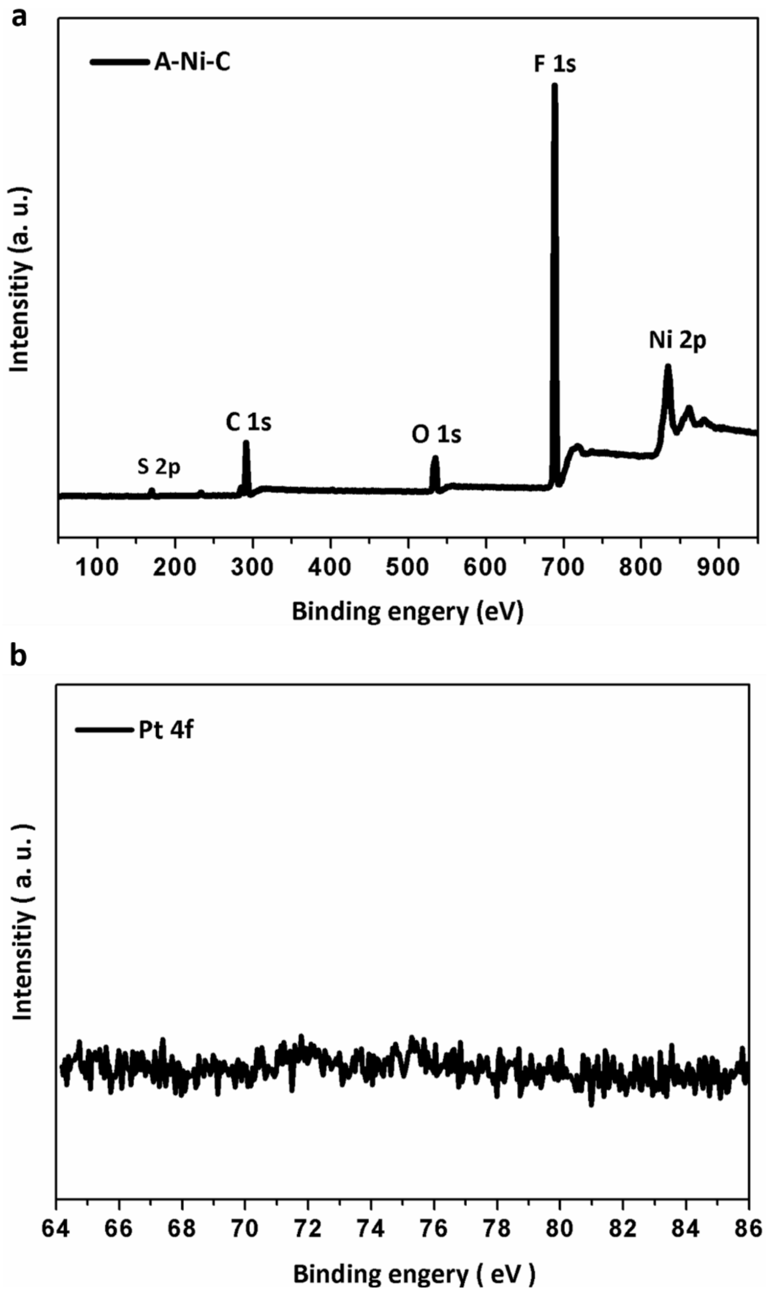

Supplementary Figure 6. (**a**) XPS spectrum of A-Ni-C sample scraped off the GCE. It contains Ni, C, F, S and O elements, without any Pt impurity. (**b**) XPS spectrum of Pt 4f core level region. No Pt was detected after activation treatment.

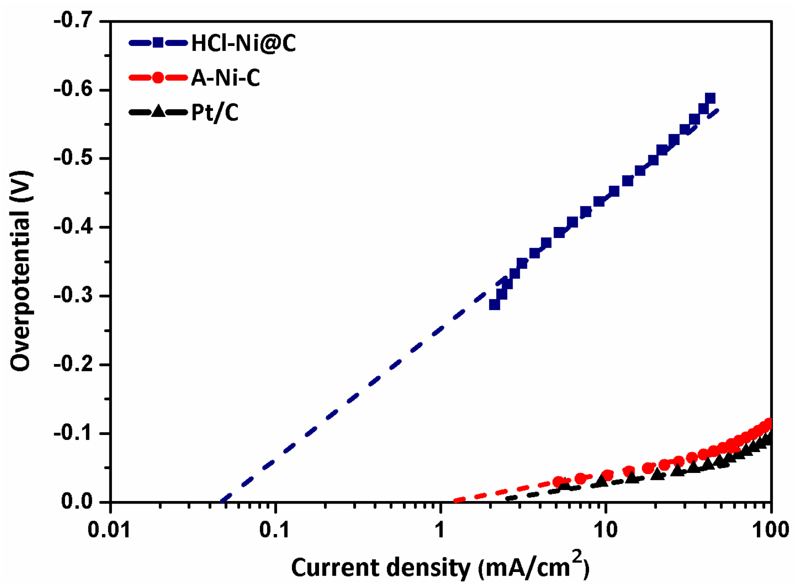

Supplementary Figure 7. Exchange current densities of HCl-Ni@C, A-Ni-C and Pt/C by using extrapolation method. In this way, j_0 of 0.05, 1.2 and 2.5 mA/cm^2 for HCl-Ni@C, A-Ni-C and Pt/C, respectively, were determined.

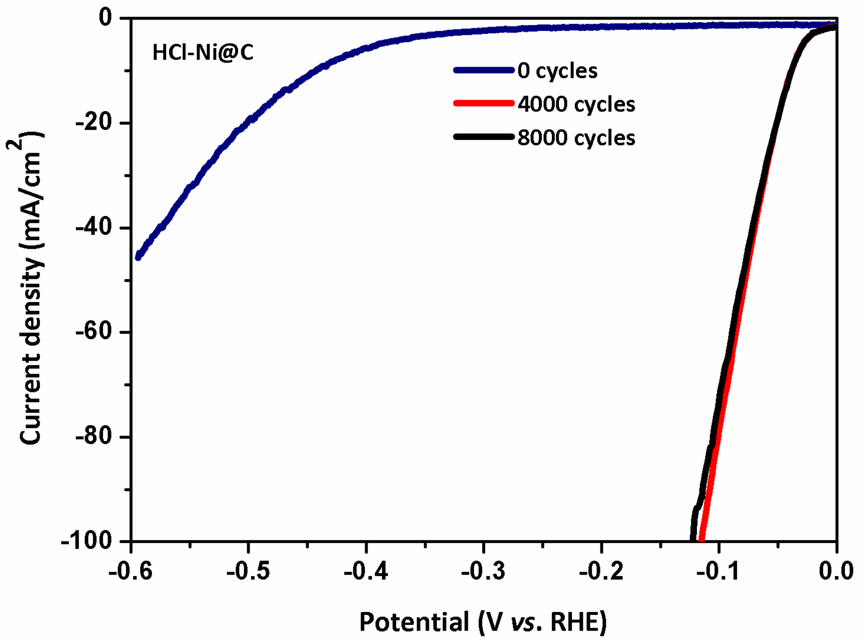

Supplementary Figure 8. Initial and post-potential-cycling LSV curves of HCl-Ni@C after 4000 and 8000 cycles between + 905 mV and - 95 mV (*vs*. RHE) at 100 mV/s were recorded. Whereas HCl-Ni@C reached the best and stable performance after about 4000 cycles, with slightly decrease in current density for the consecutive CV scans.

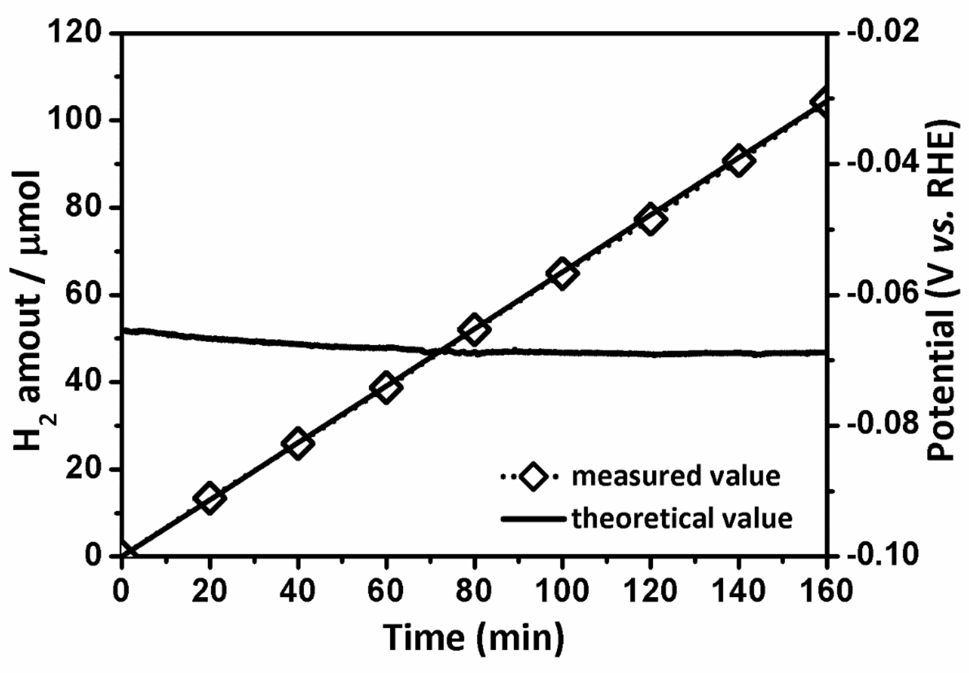

Supplementary Figure 9. Efficiency of hydrogen production over the A-Ni-C catalyst. Conditions: the working electrode is A-Ni-C decorated GCE of 3 mm in diameter, and the galvanostatic test was conducted at the current of 2.1 mA.

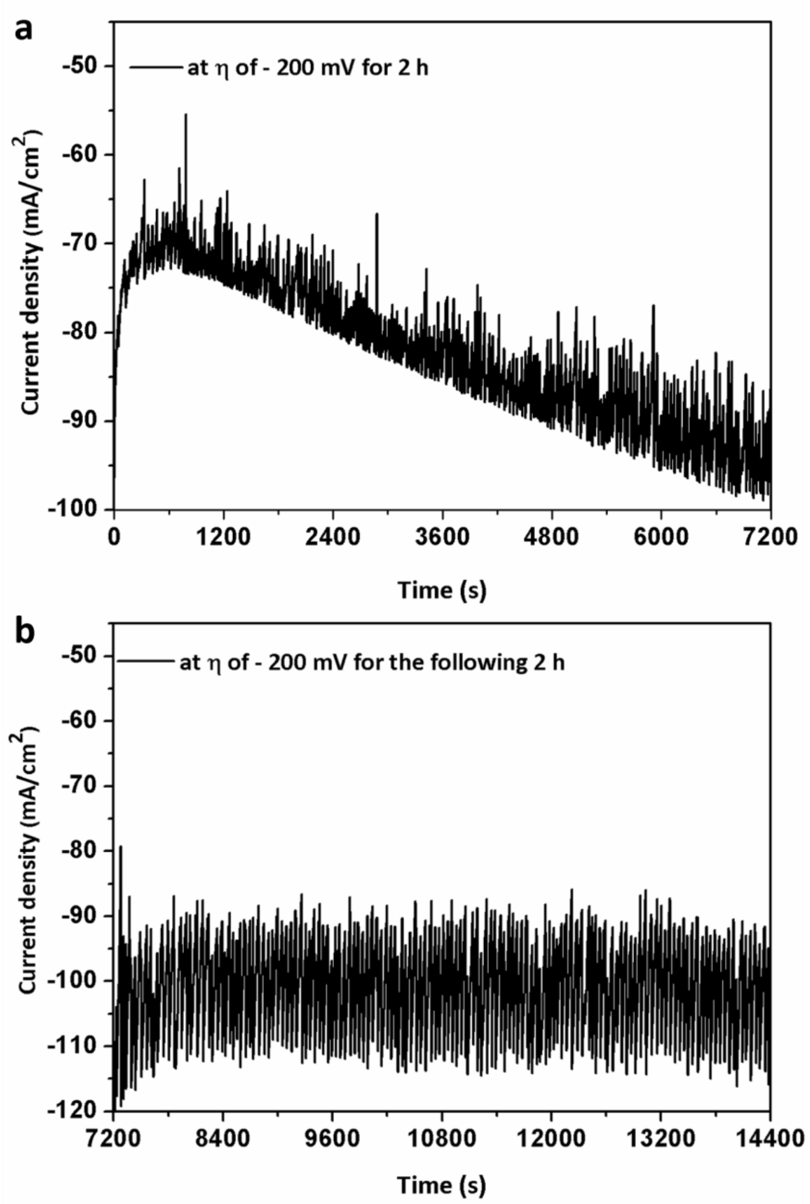

Supplementary Figure 10. (**a**, **b**) Chronoamperometric curves of Ni@C for (**a**) 2 h at η = - 200 mV and (**b**) the following 2 h at η = - 200 mV. The j gradually increased in the first 2 h and levelled out in the next 2 h.

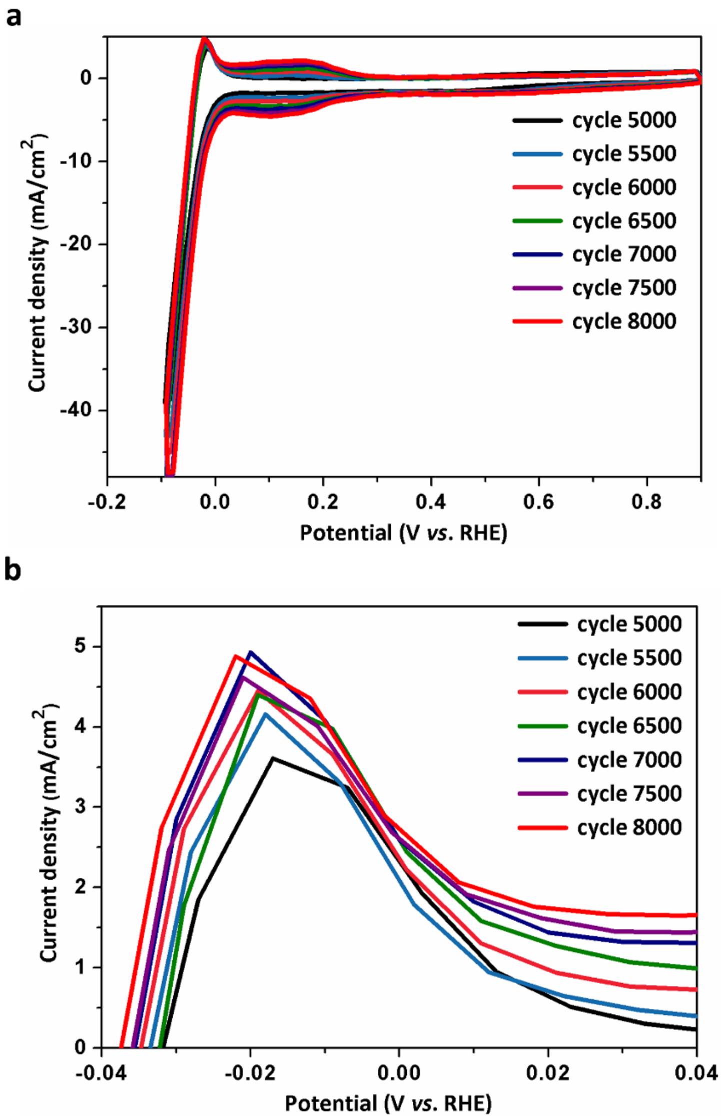

Supplementary Figure 11. (**a**) Cyclic voltammograms of the Ni-C catalyst (cycle 5000, cycle 5500, cycle 6000, cycle 6500, cycle 7000, cycle 7500 and cycle 8000 were selected), suggesting the activity for HER has levelled out. (**b**) Oxidation peaks of the Ni-C catalyst in different cycles. The intensities of the peaks changed very little during the consecutive CV treatment.

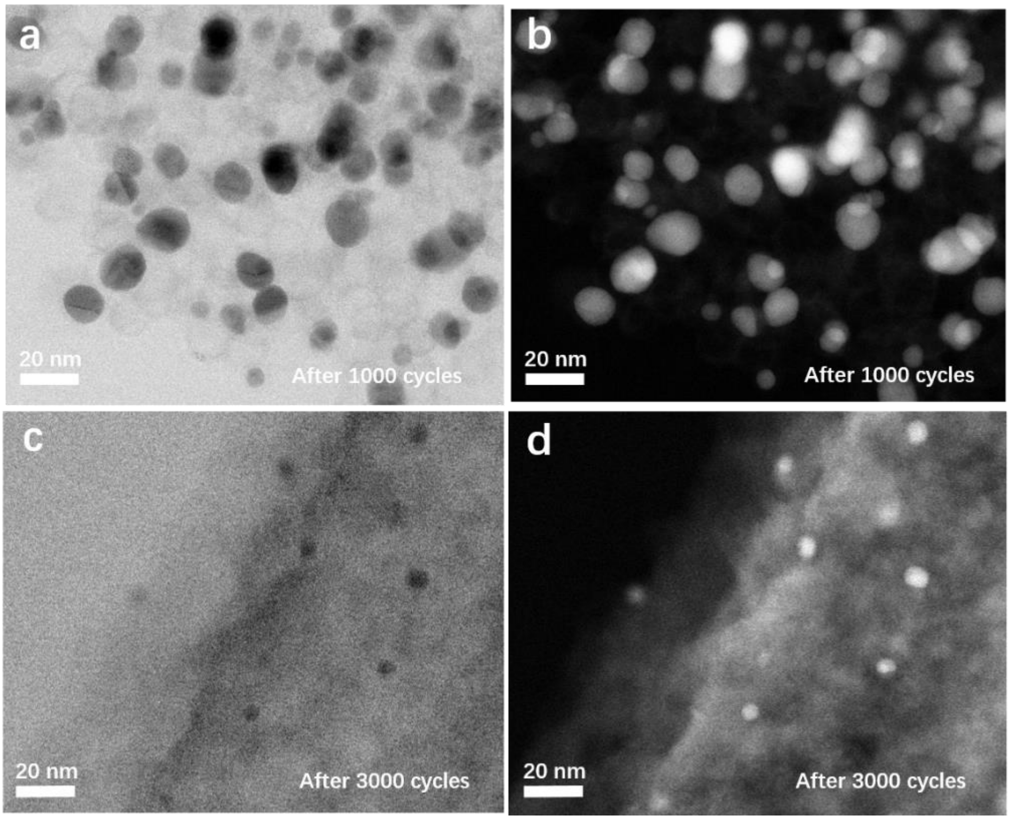

Supplementary Figure 12. A typical series STEM images of different samples of A-Ni-C after different cycles' activation (Cycle 1000 and cycle 3000 were selected). (**a**, **c**) BF STEM images and (**b**, **d**) corresponding HAADF STEM images of the same area, proving the Ni particles encapsulated in graphitized carbon shell gradually dissolved in the acid during the activation process.

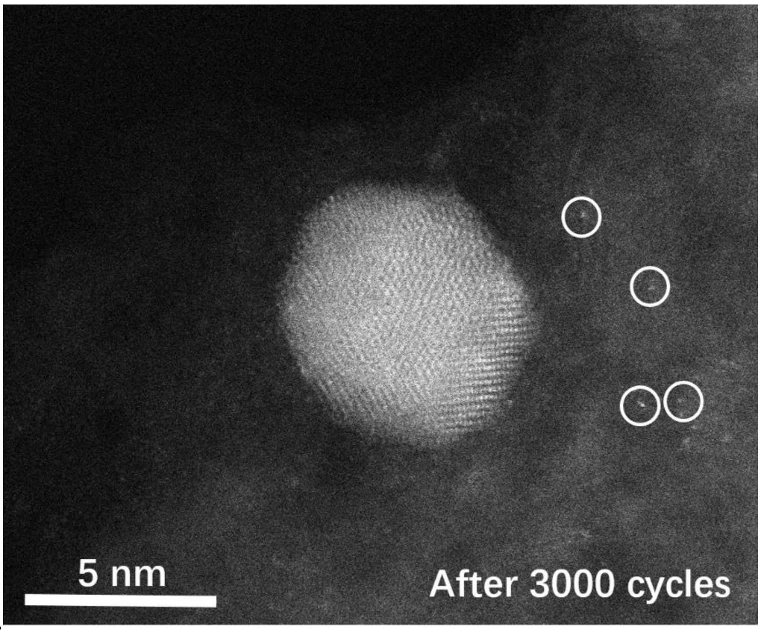

.

Supplementary Figure 13. HAADF STEM images of A-Ni-C after 3000 cycles' activation, showing the existence of single Ni atoms around the Ni particle. Considering that the elements of N, F and S have a smaller atomic number compared with Ni, thus the single atoms of these elements can't be observed as the bright spots in such an environment.

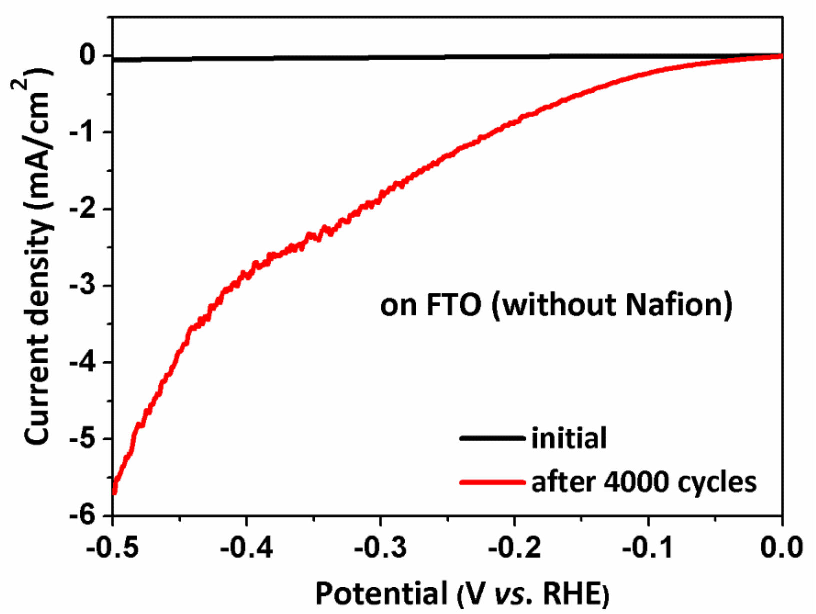

Supplementary Figure 14. LSV curves of the HCl-Ni@C samples before and after CV activation. Note: the catalysts were spin-coated onto the FTO substrate; the performances were not *iR*-corrected. Considering the poor electronic conductivity of the FTO substrate and the weak adhesion between the catalysts and the FTO substrate, the current density of the A-Ni-C is not as high as that of the catalysts deposited on GCE with Nafion as the binder. However, the activation process of the Ni-C catalysts still occurred without Nafion as the binder.

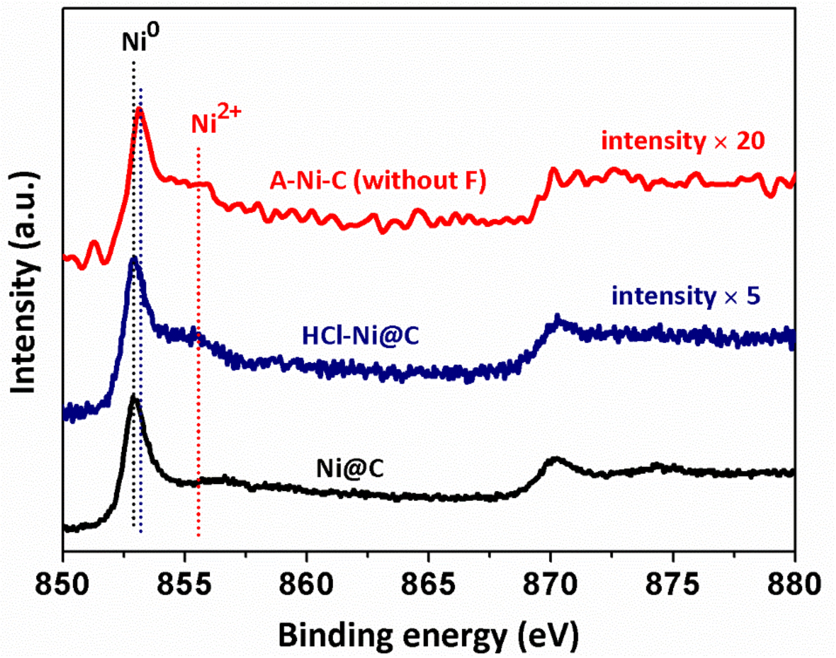

Supplementary Figure 15. XPS spectra of Ni 2p peaks of the Ni@C, HCl-Ni@C and A-Ni-C samples. Note: the binding energy of the Ni $2p_{3/2}$ peak in the A-Ni-C sample is about 853.2 eV, which is a little bit higher than that in the Ni@C and HCl-Ni@C samples (852.8 eV), indicating the possible existence of Ni-C bonding.

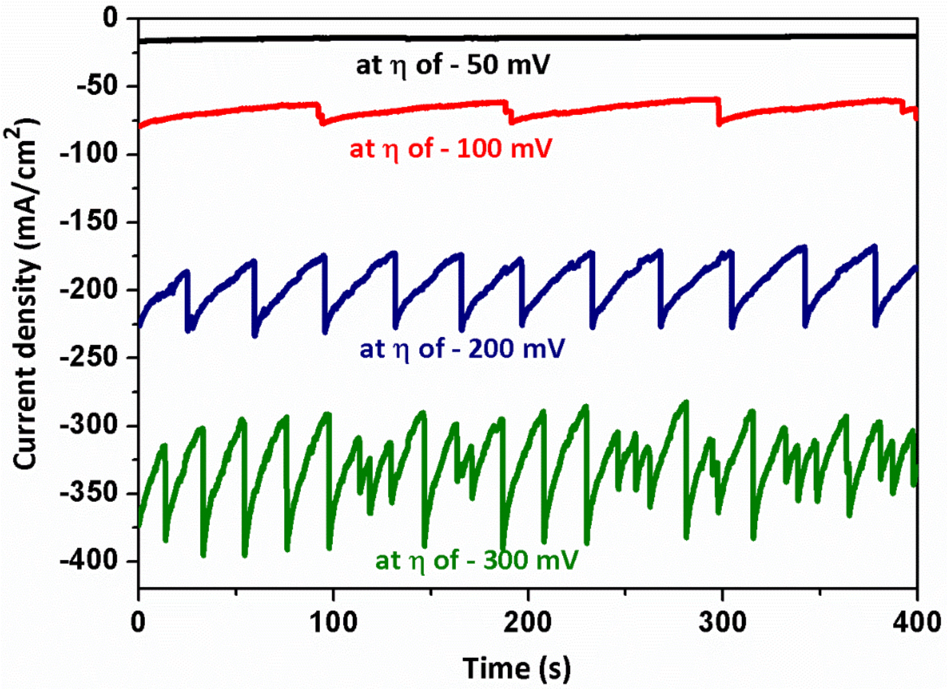

Supplementary Figure 16. Chronoamperometric curves for the titanium (Ti) foil (geometric area: 0.25 cm^2) decorated with HCl-Ni@C (with the same mass loading of 0.283 mg/cm^2 as GC electrode) after CV activation treatment, corresponding to hydrogen generation in Supplementary Movie 1.

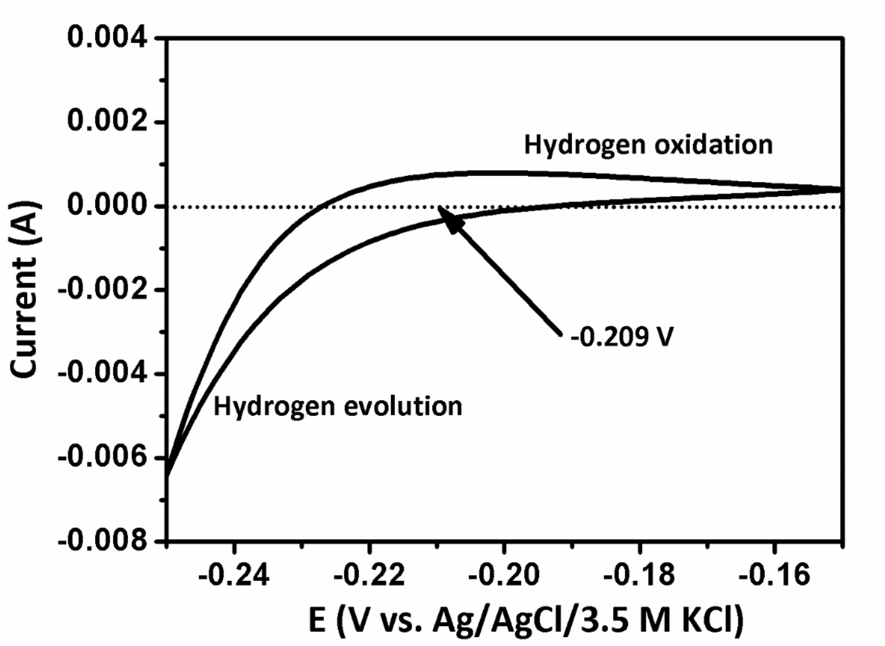

Supplementary Figure 17. The calibration of Ag/AgCl/3.5 M KCl reference electrode with respect to RHE. Thus, in 0.5 M H_2SO_4, $E_{RHE} = E_{Ag/AgCl} + 0.209$ V.

Supplementary Table 1. The percentage for C, O, N, S, F and Ni elements in A-Ni-C by XPS analysis.

Elements	C	O	N	S	F	Ni
Percentage (at.%)	35.71	14.41	0.79	2.05	46.10	0.94

Supplementary Table 2. The Ni and Pt contents in the catalysts by ICP analysis.

Sample	Ni (wt.%)	Pt (wt.%)
Ni@C	85.0	/
HCl-Ni@C	5.3	/
A-Ni-C	1.5	/

Supplementary Table 3. Comparison of selected state-of-art non-Pt HER electrocatalysts in acidic aqueous media

([a] catalysts directly grown on the conductive substrate, [b] not *iR*-corrected).

Catalyst (mg/cm^2)	Current density (j, mA/cm^2)	Corresponding overpotential (η, mV)	Tafel slope (mV/dec)	Exchange current density (j_0, mA/cm^2)	Ref.
A-Ni-C (0.283)	- 10	- 34[b]	41[b]	1.2	this work
	- 20	- 48[b]			
	- 100	- 112[b]			
double-gyroid MoS_2/FTO[a] (0.06)	- 2	- 190	50	6.9×10^{-4}	1
exfoliated MoS_2 NS	- 10	- 195	54	/	2
defect-rich MoS_2 (0.285)	- 13	- 200	50	8.91×10^{-3}	3
oxygen-incorporated MoS_2 NS (0.285)	- 10	~ - 180	55	1.26×10^{-2}	4
MoS_2/RGO (0.285)	- 10	~ - 150	41	/	5
MoS_x/NCNT (0.285)	- 10	- 110[b]	40[b]	3.31×10^{-2}	6
MoS_x/graphene/Ni foam[a] (5.01)	- 10	- 141	42.8	/	7
	- 100	- 263			
exfoliated WS_2 NS (0.1×10^{-3}-0.2×10^{-3})	- 10	~ - 240[b]	60	2.0×10^{-2}	8
WS_2 nanoflakes (0.35)	- 13	~ - 140	48	/	9
$CoSe_2$ NP/CP[a] (2.5-3.0)	- 10	- 139	42.1	$(4.9 \pm 1.4)\times 10^{-3}$	10
	- 100	- 184			
CoS_2 NW/Graphite[a] (1.4-2.0)	- 10	- 145	51.6	2.8×10^{-3}	11
nanoporous Mo_2C NWs (0.21)	- 60	- 200	54	/	12
Mo_2C/CNT (2.0)	- 10	- 152	55.2	1.4×10^{-2}	13
Mo_2C/CNT-graphene (0.65-0.67)	- 10	- 130	58	6.2×10^{-2}	14
$NiMoN_x$/C (0.25)	- 2	- 170	35.9	0.24	15
$Co_{0.6}Mo_{1.4}N_2$ (0.24)	- 10	- 200	/	0.23	16
Ni_2P hollow NPs/Ti (1.0)	- 10	- 116	46	3.3×10^{-2}	17
	- 100	- 180	81	0.49	
CoP/Ti (2.0)	- 20	- 85	50	0.14	18
CoP/CC (0.92)	- 10	- 67	51	0.29	19
	- 20	- 100			
interconnected network of MoP NPs (0.36)	- 10	- 125	54	0.086	20

**MoP | S/Ti[d] (3.0)**	- 20	- 78	~50	0.57	**21**
	- 100	- 120			
**FeP NA/Ti[a] (3.2)**	- 10	- 55	38	0.42	**22**
	- 100	- 127			
**FeP NPs/Ti (1.0)**	- 10	- 50	37	0.43	**23**
	- 20	- 61			
**C_3N_4@NG (0.1)**	- 10	- 240	51.5	3.5×10^{-4}	**24**
**Co-NRCNTs (0.28)**	- 10	- 260	69	1.0×10^{-2}	**25**
**FeCo@NCNTs-NH (0.32)**	- 10	~ - 270	74	/	**26**

Supplementary References

1. Kibsgaard, J., Chen, Z., Reinecke, B. N. & Jaramillo, T. F. Engineering the surface structure of MoS_2 to preferentially expose active edge sites for electrocatalysis. *Nature Mater.* **11**, 963-969 (2012).
2. Lukowski, M. A., Daniel, A. S., Meng, F., Forticaux, A., Li, L. & Jin, S. Enhanced hydrogen evolution catalysis from chemically exfoliated metallic MoS_2 nanosheets. *J. Am. Chem. Soc.* **135**, 10274-10277 (2013).
3. Xie, J. *et al.* Defect-rich MoS_2 ultrathin nanosheets with additional active edge sites for enhanced electrocatalytic hydrogen evolution. *Adv. Mater.* **25**, 5807-5813 (2013).
4. Xie, J. *et al.* Controllable disorder engineering in oxygen-incorporated MoS_2 ultrathin nanosheets for efficient hydrogen evolution. *J. Am. Chem. Soc.* **135**, 17881-17888 (2013).
5. Li, Y. *et al.* MoS_2 nanoparticles grown on graphene: an advanced catalyst for the hydrogen evolution reaction. *J. Am. Chem. Soc.* **131**, 7296-7299 (2011).
6. Li, D. J. *et al.* Molybdenum sulfide/N-doped CNT forest hybrid catalysts for high-performance hydrogen evolution reaction. *Nano Lett.* **14**, 1228-1233 (2014).
7. Chang, Y-H. *et al.* Highly efficient electrocatalytic hydrogen production by MoS_x grown on graphene-protected 3D Ni foams. *Adv. Mater.* **25**, 756-760 (2013).
8. Voiry, D. *et al.* Enhanced catalytic activity in strained chemically exfoliated WS_2 nanosheets for hydrogen evolution. *Nature Mater.* **12**, 850-855 (2013).
9. Cheng, L. *et al.* Ultrathin WS_2 nanoflakes as a high-performance electrocatalyst for the hydrogen evolution reaction. *Angew. Chem. Int. Ed.* **53**, 7860-7863 (2014).
10. Kong, D., Wang, H., Lu, Z. & Cui, Y. $CoSe_2$ nanoparticles grown on carbon fiber paper: an efficient and stable electrocatalyst for hydrogen evolution reaction. *J. Am. Chem. Soc.* **136**, 4897-4900 (2014).
11. Faber, M. S. *et al.* High-performance electrocatalysis using metallic cobalt pyrite (CoS_2) micro- and nanostructures. *J. Am. Chem. Soc.* **136**, 10053-10061 (2014).
12. Liao, L. *et al.* A nanoporous molybdenum carbide nanowire as an electrocatalyst for hydrogen evolution reaction. *Energy Environ. Sci.* **7**, 387-392 (2014).
13. Chen, W. –F. *et al.* Highly active and durable nanostructured molybdenum carbide electrocatalysts for hydrogen production. *Energy Environ. Sci.* **6**, 943-951 (2013).
14. Youn, D. H. *et al.* Highly active and stable hydrogen evolution electrocatalysts based on molybdenum compounds on carbon nanotube–graphene hybrid support. *ACS Nano* **8**, 5164-5173 (2014).
15. Chen, W. -F. *et al.* Hydrogen-evolution catalysts based on non-noble metal nickel-molybdenum nitride nanosheets. *Angew. Chem. Int. Ed.* **51**, 6131-6135 (2012).

16. Cao, B., Veith, G. M., Neuefeind, J. C., Adzic, R. R. & Khalifah, P. G. Mixed close packed cobalt molybdenum nitrides as non-noble metal electrocatalysts for the hydrogen evolution reaction. *J. Am. Chem. Soc.* **135**, 19186-19192 (2013).
17. Popczun, E. J. *et al.* Nanostructured nickel phosphide as an electrocatalyst for the hydrogen evolution reaction. *J. Am. Chem. Soc.* **135**, 9267-9270 (2013).
18. Popczun, E. J., Read, C. G., Roske, C. W., Lewis, N. S. & Schaak, R. E. Highly active electrocatalysis of the hydrogen evolution reaction by cobalt phosphide nanoparticles. *Angew. Chem. Int. Ed.* **53**, 5427-5430 (2014).
19. Tian, J., Liu, Q., Asiri, A. M. & Sun, X. Self-supported nanoporous cobalt phosphide nanowire arrays: an efficient 3D hydrogen-evolving cathode over the wide range of pH 0–14. *J. Am. Chem. Soc.* **136**, 7587-7590 (2014).
20. Xing, Z., Liu, Q., Asiri, A. M. & Sun, X. Closely interconnected network of molybdenum phosphide nanoparticles: a highly efficient electrocatalyst for generating hydrogen from water. *Adv. Mater.* **26**, 5702-5707 (2014).
21. Kibsgaard, J. & Jaramillo, T. F. Molybdenum phosphosulfide: an active, acid-stable, earth-abundant catalyst for the hydrogen evolution reaction. *Angew. Chem. Int. Ed.* **53**, 14433-14437 (2014).
22. Jiang, P. *et al.* A cost-effective 3D hydrogen evolution cathode with high catalytic activity: FeP nanowire array as the active phase. *Angew. Chem. Int. Ed.* **53**, 12855-12859 (2014).
23. Callejas, J. F. *et al.* Electrocatalytic and photocatalytic hydrogen production from acidic and neutral-pH aqueous solutions using iron phosphide nanoparticles. *ACS Nano* **8**, 11101-11107 (2014).
24. Zheng, Y. *et al.* Hydrogen evolution by a metal-free electrocatalyst. *Nature Commun.* **5**, 3783 (2014).
25. Zou, X. *et al.* Cobalt-embedded nitrogen-rich carbon nanotubes efficiently catalyze hydrogen evolution reaction at all pH values. *Angew. Chem. Int. Ed.* **53**, 4372-4376 (2014).
26. Deng, J. *et al.* Highly active and durable non-precious-metal catalyst encapsulated in carbon nanotubes for hydrogen evolution reaction. *Energy Environ. Sci.* **7**, 1919-1923 (2014).
